# Supplementary material for: Caffeine ingestion improves morning neuromuscular performance to evening levels in healthy females
Source: PLoS One. 2026 May 7;21(5):e0336199. doi: 10.1371/journal.pone.0336199 (PMC13152181; doi:10.1371/journal.pone.0336199)
Supplement: Attachment — Submitted filename: Response to Reviewer.docx [file pone.0336199.s001.docx]

**We thank the Academic Editor and both reviewers for their careful evaluation of our manuscript and for their constructive and insightful comments. We have revised the manuscript accordingly and believe that these changes have strengthened the clarity, methodological rigour, and overall quality of the work. A detailed, point-by-point response to each comment is provided below. Reviewer comments are reproduced in black, and our responses are provided in bold red text beneath each comment. All changes made to the manuscript are reflected in the revised version.**

Reviewer #1: This study is relevant to the scientific community specifically in the area of exercise physiology and health.

**We thank Reviewer #1 for their constructive feedback and for recognising the relevance of the study to exercise physiology and health research. All comments are addressed individually below.**

Therefore, some considerations regarding the study methodology were made:
1) The distribution of the sample into groups did not include an isolated control group. Since each participant was controlled by themselves, at what point were the data relating to the control phase of the study collected? Include this data in the statistics for comparison with the other groups.

**We appreciate this comment and have clarified the within-subject control structure of the study. As a repeated-measures crossover design was employed, an isolated control group was not required. The morning placebo (AM_PLAC_) and evening placebo (PM_PLAC_) conditions served as control phases, enabling direct comparison of time-of-day effects and caffeine ingestion within the same participants. Data from all placebo and caffeine conditions were included in the statistical analyses and are presented in Table 1 and Figures 2–4. New text has also been added to the ‘Study design and Supplementation section in the methods to help with clarity.**

2) It was reported that there were 3 evaluation sessions. Was there a data collection session solely for baseline measurements? If this collection occurred in the same performance session, what was the interval between the end of the baseline measurement collection and the beginning of the performance data collection?

**Baseline measurements were collected at the start of each experimental session and not during a separate visit. These measurements were immediately followed by a standardised warm-up, after which performance testing commenced. Wording has also been changed in the experimental protocol section of the methods to ensure clarity of timing between baseline collection and beginning of warm-up and experimental procedures.**

3) Provide a reference regarding the collection of tympanic temperature. Why was this measurement chosen?

**Tympanic temperature was selected as a non-invasive and practical surrogate of core temperature, suitable for repeated measurements across multiple experimental sessions. Tympanic thermometry is widely used in exercise and circadian research and has been shown to reflect changes in core body temperature under resting and exercise conditions. Appropriate reference (PMID: 12427049) has now been added along with new text in the experimental protocol section of the methods to support the validity and rationale for this methodological choice.**

4) Regarding electromyography, how many electrodes were used? Why was the vastus medialis muscle chosen? Why was only this muscle chosen and not others of the quadriceps femoris?
**Surface electromyography was recorded using bipolar electrodes placed over a single muscle (vastus medialis) in accordance with SENIAM guidelines. The vastus medialis was selected due to its major contribution to knee extensor torque during isometric contractions, its superficial anatomy allowing reliable surface EMG recordings, and its frequent use in studies examining neuromuscular activation and fatigue of the knee extensors. Recording EMG from a single representative quadriceps muscle is a common and accepted approach when the primary aim is to assess relative changes in neuromuscular activation across conditions. Limiting recordings to one muscle also reduces cross-talk, improves signal quality, and enhances repeatability across sessions. Appropriate justification and references have now been added to the Neuromuscular testing section in the Methods.**

5) What were the inclusion and exclusion criteria for the sample?

**We thank the reviewer for highlighting the need for explicit reporting. Inclusion and exclusion criteria have now been clearly specified in the Subjects section of the methods, including health status, caffeine consumption, menstrual cycle considerations, and exercise- and medication-related restrictions.**

6) How was randomization performed?

**We thank the reviewer for highlighting the importance of clearly reporting the testing sequence, which has helped improve the methodological transparency of the manuscript. The order of experimental conditions was not randomised; instead, a fixed testing sequence was deliberately employed to accommodate the circadian nature of the study design and to minimise potential caffeine carryover effects between sessions. Specifically, the evening placebo condition was completed separately from the morning trials, and the morning caffeine condition was performed after the morning placebo condition to avoid residual caffeine influencing placebo measurements. We believe this fixed sequence was an appropriate and conservative design choice for addressing the study aims. The testing order has now been explicitly clarified in the study design and supplementation section of the Methods, and any wording implying randomisation has been removed including the title. This aspect of the design has also been acknowledged in the Limitations section.**

7) What was the fitness level of the participants? Had the sample previously participated in a strength training program?

**We thank the reviewer for highlighting the importance of describing participant fitness and training background. Participants were recreationally active but not engaged in structured resistance or strength training programmes, and none had prior experience with isometric strength testing. This information has now been explicitly added to the Subjects section of the Methods.**

8) Why wasn't the sample's BMI calculated for comparison purposes?

**We thank the reviewer for this observation. Body mass was recorded and peak torque was normalised to body mass, which we considered more physiologically relevant for interpreting neuromuscular performance outcomes than BMI. BMI does not account for body composition and is less informative for strength-related measures in healthy, non-obese participants. As such, BMI was not included as a primary descriptive or comparative variable. This rationale has now been clarified in the statistical analysis section of the Methods.**

9) Include the assessment of neuromuscular function under fatigue conditions in the last paragraph of the introduction.

**We thank the reviewer for this helpful suggestion. The final paragraph of the Introduction has now been revised to explicitly include the assessment of neuromuscular function under fatigued conditions, in line with the experimental design and outcomes measured in the study.**

10) The graphs should be self-explanatory. I suggest creating a legend for each group and for each symbol used.

**We thank the reviewer for this suggestion. All figures were designed to be stand-alone and include condition labels, colour coding, and symbol definitions within the legends. To further improve clarity, the figure legends have been slightly expanded to explicitly describe group coding and the meaning of individual data points and bar shading. No changes to the data or graphical presentation were required.**

11) What were the results of the effect sizes of the research?

**We thank the reviewer for this comment. Effect sizes (η² and partial η²) were calculated for all main and interaction effects and are reported in Table 1 alongside inferential statistics. These values indicate moderate to large effects across the primary neuromuscular outcomes, supporting the practical relevance of the findings. To improve clarity, this has now been briefly highlighted in the Results section were Table 1 is introduced.**

14) In the Discussion, you should compare the results found with studies already conducted on the topic and not just describe your results. This is the part of the manuscript where you should cite other authors of related studies for comparison purposes.

**We thank the reviewer for this comment. The Discussion has been revised to more explicitly compare the present findings with previously published studies, highlighting areas of agreement and divergence with existing literature, particularly with respect to task specificity, effect magnitude, and neuromuscular mechanisms.**

Reviewer #2: Thank you for the opportunity to review this interesting and timely manuscript examining whether acute caffeine ingestion can restore morning neuromuscular performance to evening levels in healthy young females. The topic is relevant to exercise physiology, chronobiology, sport science, and women’s health research, and the manuscript is generally clear, well written, and logically structured. Importantly, it addresses a recognised gap in the literature: the underrepresentation of female participants in caffeine research and the complexity introduced by menstrual cycle-related hormonal fluctuations.
The study provides valuable data on the interaction between caffeine, time-of-day, and neuromuscular function, offering potentially meaningful practical implications for morning performance optimisation. The experimental design is conceptually sound, the measurements are appropriate for the research question, and the authors articulate their findings coherently. The manuscript therefore has clear potential for publication; however, several methodological and reporting issues reduce the current level of rigour and require clarification or expansion before the manuscript can be considered suitable for PLOS Aging and Health.
Below I outline my comments in detail, separating general concerns from specific points requiring revision. The study demonstrates adequate originality for the journal, particularly in its focus on young women—a population that remains understudied in caffeine-related performance research. The chronobiological framing adds conceptual value and the results complement existing knowledge regarding diurnal variations in strength, endurance, and neuromuscular activation.
Nevertheless, certain aspects limit the broader impact expected for a publication in PLOS Aging and Health: chiefly the small sample size, limited generalisability beyond young adults, and the narrow task specificity (isometric knee extensor MVC and endurance tests). Strengthening the rationale and contextualisation in terms of lifespan health and sex-specific physiology would enhance the manuscript’s relevance for readers across the ageing and health sciences spectrum.
Importantly, although the study meets ethical standards and uses recognised methodologies, several key elements require more detailed reporting to satisfy the journal’s requirement for high methodological rigour and substantial evidence supporting the conclusions.

**We thank Reviewer #2 for their thoughtful, detailed, and constructive evaluation of our manuscript. We appreciate their positive assessment of the study’s relevance, conceptual framing, and contribution to addressing the underrepresentation of females in caffeine research. We also acknowledge the reviewer’s important points regarding methodological transparency, reporting clarity, and broader contextualisation within ageing and health sciences. In response, we have revised the manuscript to provide additional methodological detail, clearer justification of design choices, and strengthened discussion of the findings in relation to sex-specific physiology and lifespan health considerations. Each comment is addressed individually below.**

I recommend moderate revisions.
The manuscript states that 13 healthy young females participated and were classified as light caffeine consumers. While this provides a general notion of the participant pool, essential details regarding the physical activity level and training status are missing. These factors significantly affect neuromuscular performance, caffeine responsiveness, fatigue tolerance, and task familiarity. Without them, generalisability is unclear and between-participant variability cannot be fully contextualised.
Recommended revision:
Please provide explicit information on the participants’ habitual physical activity, including estimated weekly volume, type of exercise (aerobic, resistance, recreational sport), and whether any had experience with isometric or strength testing. This information should also be incorporated into the inclusion criteria.
Additionally, please list inclusion and exclusion criteria explicitly. At present, these must be inferred (health status, menstrual phase, caffeine intake, etc.), but explicit statements would improve transparency and reproducibility.

**We thank the reviewer for highlighting the importance of clearly characterising participants’ physical activity and training background. In response, we have expanded the Subjects section of the methods to explicitly describe habitual physical activity level and prior resistance or isometric exercise experience. Participants were recreationally active but not engaged in structured strength or resistance training programmes, and none had prior experience with isometric knee extensor testing beyond familiarisation. In addition, inclusion and exclusion criteria have now been explicitly listed in the Subjects section, including health status, caffeine consumption, menstrual cycle considerations, physical activity level, and medication or supplement use. These revisions improve transparency, reproducibility, and contextualisation of inter-individual variability.**

The manuscript notes that testing occurred in the mid-follicular phase (~days 10–15), which is appropriate given the need to minimise hormonal variation. However, it is not stated how this phase was determined. Self-report, cycle diary, ovulation predictors, or hormonal assays each have different levels of reliability. Moreover, it is not indicated whether hormonal contraceptive users were excluded, even though contraceptives may substantially affect caffeine metabolism (via modulation of CYP1A2), thermoregulation, substrate utilisation, and neuromuscular efficiency.
Given the known importance of hormonal dynamics in caffeine studies involving women, these omissions weaken the methodological transparency and must be addressed.

Recommended revision:
• Specify explicitly how menstrual cycle phase was determined (e.g., self-reported cycle day, tracking apps, hormonal confirmation).
• State whether hormonal contraceptive use was an exclusion criterion. If not, please clarify whether any participants used contraceptives and include this as a limitation.
This is essential for reproducing the study and interpreting intra- and inter-participant variability.

**We thank the reviewer for highlighting the importance of clearly reporting menstrual cycle determination and contraceptive status. Menstrual cycle phase was determined using self-reported cycle length and day of cycle, confirmed verbally at each visit, with all testing scheduled during the mid-follicular phase (~days 10–15). This approach has been explicitly stated in the Subjects section of the Methods. Hormonal contraceptive use was an exclusion criterion, and no participants were using oral or other hormonal contraceptives. This has now been clearly reported in the exclusion criteria. These additions improve methodological transparency and support reproducibility and interpretation of inter-individual variability.**

The manuscript instructs participants to abstain from caffeine, alcohol, smoking, and strenuous exercise for 48 hours prior to each trial. However, the experimental protocol involves three sessions on consecutive days, making it impossible for participants to meet this requirement before each session unless the authors intended abstinence to begin 48 hours before the first session and continue throughout.
Furthermore, because caffeine was administered in only one condition, and sessions occurred within a short time frame, it is crucial to clarify whether:
• the caffeine session was always placed last, or
• conditions were randomised, in which case absence of a washout period becomes a significant methodological concern.
This requires explicit correction.
Recommended revision:
• Clarify whether abstinence was continuous throughout the three days rather than reset before each session.
• Specify the order of the three conditions (Morning Placebo, Morning Caffeine, Evening Placebo).
• If randomised, acknowledge that a washout period longer than 24 hours may be required to eliminate residual caffeine effects, especially given inter-individual differences in CYP1A2 activity.
• Discuss this explicitly in the Limitations section.
This is important because caffeine’s half-life, combined with individual metabolic variability, may lead to carry-over effects.

**We thank the reviewer for highlighting this important point regarding abstinence instructions, session order, and potential caffeine carry-over effects. Abstinence from caffeine, alcohol, smoking, and strenuous exercise was continuous throughout the three consecutive testing days and began at least 48 hours prior to the first experimental session, rather than being reset before each visit. This has now been clarified in the Methods section.
The order of experimental conditions was fixed and not randomised. Specifically, participants completed the evening placebo trial first, followed by the morning placebo trial, and finally the morning caffeine trial. This fixed sequence was deliberately chosen to preserve circadian timing and to minimise potential carry-over effects of caffeine on placebo conditions.
The testing order and abstinence protocol have now been explicitly stated in the Methods section, and the lack of randomisation and potential order effects are acknowledged in the Limitations section. These revisions improve transparency and address concerns regarding residual caffeine effects and inter-individual variability in caffeine metabolism.**

The use of a single-blind design is stated, but the manuscript does not reflect on the implications. Given that caffeine often produces noticeable physiological sensations (increased alertness, heart rate changes, etc.), the blinding integrity may have been compromised.
Furthermore, no assessment of blinding efficacy (e.g., asking participants to guess their condition) is reported. This weakens interpretability, particularly for subjective outcomes such as perceived exertion.
Recommended revision:
Please discuss the limitations of the single-blind design more explicitly and consider adding a note in the Limitations section regarding potential expectancy bias. While not fatal to the study, acknowledging this improves transparency.

**We thank the reviewer for raising this important point. The study employed a single-blind design, and we agree that the physiological sensations associated with caffeine ingestion may have partially compromised blinding integrity. No formal assessment of blinding efficacy (e.g., condition guessing) was performed. This limitation has now been more explicitly acknowledged in the Limitations section, including the potential influence of expectancy effects on subjective outcomes such as perceived exertion. While this does not invalidate the findings, we agree that transparency regarding this issue improves interpretability.**

The choice of 6 mg/kg is common in ergogenic research and the ingestion timing (45 minutes pre-test) aligns with established pharmacokinetics. However, providing a brief justification in the Methods section, referencing plasma peak timing, would strengthen methodological clarity.

**We thank the reviewer for this suggestion. The Methods section has now been revised to make the rationale for the selected caffeine dose (6 mg·kg⁻¹) and ingestion timing (45 min pre-test) more explicit. Specifically, we clarify that this timing was chosen to coincide with peak plasma caffeine concentrations, which typically occur 30–60 minutes post-ingestion, as supported by established pharmacokinetic evidence.**

Additionally, in the Practical Applications or Discussion section, please briefly acknowledge that this dose is relatively high and may not be suitable for all individuals. This will help meet the journal’s requirement for clear utility for the broader community, especially given differences in caffeine sensitivity among women.

**We thank the reviewer for this suggestion. A brief acknowledgement has now been added to the Discussion highlighting that the selected caffeine dose (6 mg·kg⁻¹) is relatively high and may not be suitable for all individuals, particularly given inter-individual variability in caffeine sensitivity. We also emphasise that lower doses (3–6 mg·kg⁻¹) may be more appropriate depending on tolerance, thereby improving the practical relevance of the findings for a broader population.**

The use of isometric knee extensor tasks is well established, but the manuscript does not explicitly justify this choice. Although the rationale can be inferred (large muscle group, reliable EMG signal, widely used in chronobiology and strength research), a concise explanation would improve clarity.
Recommended revision:
Add 1–2 sentences describing why knee extensors were selected, emphasising reliability, comparability to prior studies, and suitability for assessing neuromuscular responses to circadian and caffeine-related factors.

**We thank the reviewer for this suggestion. A concise justification for the selection of the knee extensor task has now been added to the Neuromuscular testing section of the Methods. Specifically, we clarify that the knee extensors were chosen due to their involvement of a large muscle group, the reliability and repeatability of isometric torque and EMG measurements, and their widespread use in studies examining circadian variation and caffeine-related neuromuscular responses. This addition improves clarity and contextualisation of the task choice.**

The manuscript does not provide a sample size justification or a power analysis. Although crossover designs reduce inter-individual variability, a formal justification is expected for publication in PLOS journals.
Recommended revision:
Include whether an a priori or post hoc power estimation was performed. If not feasible, please provide a short statement acknowledging this and discussing the implications.

**We thank the reviewer for this comment. No a priori sample size calculation was performed for the present study. This has now been explicitly stated in the Methods, and the implications of the sample size are acknowledged in the Discussion. While the repeated-measures crossover design reduces inter-individual variability and increases statistical sensitivity, we recognise that the modest sample size may limit generalisability and the detection of smaller effects. This has now been discussed as a limitation.**

This study addresses a meaningful and underexplored topic, contributes valuable data to the field, and offers potential practical relevance. With revisions addressing methodological clarity, enhanced description of participant characteristics, improved explanation of menstrual cycle control, and more cautious interpretation of mechanisms, the manuscript will meet the standards required for publication in PLOS Aging and Health.
I encourage the authors to revise the manuscript according to the points listed above. The core of the study is strong, and I believe it has good potential for publication after these improvements.

**We thank Reviewer #2 for their constructive and encouraging feedback and for recognising the contribution and potential impact of the study. We have revised the manuscript in line with the reviewer’s suggestions, including enhanced methodological clarity, more detailed reporting of participant characteristics and menstrual cycle control, and cautious interpretation of mechanistic inferences. We believe these revisions have strengthened the manuscript and improved its relevance and rigour for readers of PLOS ONE.**
