# Supplementary material for: Caffeine ingestion improves morning neuromuscular performance to evening levels in healthy females
Source: PLoS One. 2026 May 7;21(5):e0336199. doi: 10.1371/journal.pone.0336199 (PMC13152181; doi:10.1371/journal.pone.0336199)
Supplement: Attachment — Submitted filename: Response to Reviewers.docx [file pone.0336199.s002.docx]

We thank the Academic Editor and Reviewers for their continued evaluation of our manuscript and for their constructive and insightful comments. We are grateful for the opportunity to revise the manuscript further, and we appreciate the careful consideration given to our work.

We are encouraged that Reviewer #2 considers that all previous comments have been addressed and recommends the manuscript for publication. We also thank Reviewer #3 for their detailed and thoughtful feedback, particularly regarding neuromuscular assessment, methodological considerations, and interpretation of the findings. These comments have been highly valuable in further strengthening the clarity, and scientific rigour of the manuscript.

In response, we have carefully revised the manuscript to address all points raised. In particular, we have:

- clarified aspects of the experimental design and testing procedures,
- strengthened the justification and interpretation of neuromuscular outcomes,
- refined the description of menstrual cycle considerations,
- expanded the Discussion to more appropriately contextualise the findings and acknowledge limitations,
- and improved the presentation and clarity of figures as suggested by the Academic Editor.

A detailed, point-by-point response to each comment is provided below. Reviewer comments are reproduced in black, and our responses are provided in **bold red text** beneath each comment. All corresponding changes have been incorporated into the revised manuscript.

Additional Editor Comments :
Please, also consider these additional comments to improve the quality of your figures:
I suggest adjusting Figure 1 to reflect the right order and mention Day1, day2, day3.
Please reduce the bar line thickness in your figures, add lines connecting dots to see individual trajectories. Please rescale figures 3 d,e and f; and figure 4 a, b.

**We thank the Academic Editor for these helpful suggestions to improve the presentation of the figures. Figure 1 has been revised to clearly reflect the correct experimental order with explicit labelling of Day 1, Day 2, and Day 3. In addition, figure formatting has been refined by reducing bar line thickness, adding lines to connect individual data points to better visualise within-participant trajectories, and rescaling Figures 3 (panels D–F) and Figure 4 (panels A–B) to improve clarity and readability.**

Reviewer #2: The revised manuscript shows a clear and consistent effort by the authors to address the reviewers’ comments. The changes made have substantially improved the quality of the paper, particularly in terms of methodological clarity and transparency in reporting procedures and study limitations.
Although some limitations remain inherent to the study design, these are appropriately acknowledged and discussed by the authors, and do not compromise the overall coherence or scientific contribution of the work.
In this context, I consider that the manuscript meets the requirements for publication and recommend its acceptance.

**We thank Reviewer #2 for their positive and constructive feedback. We are grateful for their careful evaluation of the revised manuscript and are pleased that the changes have improved the clarity and transparency of the work. We also appreciate their recommendation for publication.**

Reviewer #3: The present manuscript investigated the effects of ingesting 6 mg·kg⁻¹ of caffeine 45 minutes prior to a morning exercise session in 13 active female participants. The aim was to determine whether caffeine could increase maximal voluntary isometric contraction (MVIC) and fatigue resistance in the morning to levels comparable to those observed in the evening. It reported that caffeine ingestion resulted in improvements in MVIC, time to exhaustion, EMG amplitude, and perceived exertion, without changes in EMG frequency characteristics or core temperature compared to placebo, with similar results to evening levels. I appreciate that this study evaluates females when the literature is historically biased on males-only studies, and I read it with interest.

**We thank Reviewer #3 for their careful reading of the manuscript and for recognising the relevance of the study. We appreciate these positive comments and address the specific concerns raised below.**

However, there are important concerns that must be addressed:
1. How do you explain that maximal torque after the task was practically identical to “pre-fatigue” when by design it should have drop below 50%? Pacing could be involved. To clarify you could consider: i) to calculate the area under the curve (total impulse) which could work for your isometric tests such as yours as performance index. Would be helpful for an interested reader also to have the longitudinal graph of the mean force contraction by contraction (should be very feasible, considering the graph should consist of max 20 points, last common stage for all participants around 40 s). That should clarify about possible pacing.

**We thank Reviewer #3 for this important and thoughtful comment. We would like to clarify that the criterion of a decline to 50% of baseline MVIC was used solely to determine task failure during the intermittent fatigue protocol, and not as an expected outcome for the post-fatigue MVIC measurement. The post-fatigue MVIC was assessed following a standardised recovery period (2 minutes), and therefore reflects the capacity to rapidly recover force production rather than the minimum force achieved during the fatigue task itself.**

**The intermittent nature of the fatigue protocol (6 s contraction, 4 s rest) may allow partial recovery between contractions, which can attenuate the decline in maximal force and contribute to the preservation of post-fatigue MVIC values. As such, the observation that post-fatigue MVIC values remained relatively high is consistent with the task design and does not indicate a deviation from the intended fatigue criterion.**

**We agree that pacing behaviour may influence performance in intermittent protocols and have now acknowledged this as a potential consideration in the Discussion. We also appreciate the reviewer’s suggestion regarding alternative analytical approaches such as area under the curve or contraction-by-contraction force profiles. While these were not included in the current analysis, they represent valuable avenues for future work and may provide additional insight into fatigue dynamics and pacing strategies in similar protocols.**

2. According to the methods section, the order was: day 1= evening placebo. Day 2 (so the morning after)= morning placebo. Day 3= morning caffeine. I wonder why the authors did not perform a MVIC before caffeine consumption, to ensure the “boost” came from caffeine consumption (as your main outcome was MVIC and it changed at pre-fatigue across conditions). This is a major methodological problem. Warm-up does not severely impact a MVIC measure (<https://doi.org/10.1016/j.jsams.2018.07.003>) so this could have been a possible way to validate the intervention.

**We thank Reviewer #3 for this important comment. We agree that incorporating an additional pre-ingestion MVIC within the same session could provide further insight into the acute effects of caffeine. However, the present study employed a within-subject, repeated-measures crossover design, in which each condition (morning placebo, morning caffeine, and evening placebo) included its own baseline MVIC assessment conducted under standardised conditions. This design allows for direct comparison of performance outcomes between conditions, with the morning placebo condition serving as the primary control for evaluating the effect of caffeine ingestion.**

**As such, the ergogenic effect of caffeine was determined by comparing performance in the morning caffeine condition relative to the matched morning placebo condition, rather than through within-session pre–post comparisons. This approach is consistent with established practice in crossover studies investigating caffeine and exercise performance, where between-condition comparisons are used to isolate treatment effects (PMID: 34291426; PMID: 29876876). As such this has been clarified in the study design and supplementation section of the methods.**

**We acknowledge that the absence of an additional pre-ingestion MVIC within the caffeine trial may limit the ability to assess within-session changes, and this has now been noted as a limitation in the Discussion. However, we believe that the current design provides a valid and controlled comparison of caffeine’s effect on neuromuscular performance relative to placebo under matched circadian conditions.**

INTRODUCTION
3. I suggest referring more specifically to maximal voluntary isometric contraction (MVIC) rather than the broader term “neuromuscular performance” in both the title and the conclusions, as the latter is less precise relative to what was actually measured. Additionally, the title could be made more specific by including the task and replacing “restores” with “enhances” or “improves”, for example:
Caffeine ingestion improves morning maximal isometric torque and performance to evening levels in healthy females during an intermittent isometric task.

**We thank Reviewer #3 for this helpful suggestion. We agree that greater specificity in terminology improves clarity. Accordingly, we have revised the title and relevant sections of the manuscript to more explicitly reflect the primary outcomes assessed, including maximal voluntary isometric contraction (MVIC) and fatigue-related measures.**

**However, we have retained the term ‘neuromuscular performance’ where appropriate, as the study assessed multiple interrelated outcomes, including MVIC, time to exhaustion, electromyographic activity, and perceived exertion, rather than a single isolated measure. We believe this terminology more accurately reflects the integrated nature of the neuromuscular responses evaluated. We have also replaced the term ‘restores’ with ‘improves’ in the title to better reflect the observed effects and to align with the reviewer’s recommendation.**

METHODS
4. L105: It would be more accurate to state that testing occurred between days 10–15 after menstruation rather than during the “mid-follicular phase.”Without objective verification (e.g., ovulation testing or hormonal measurements), it is not appropriate to assume cycle phase (<https://doi.org/10.1007/s40279-025-02189-3>). This should be clarified and discussed as a limitation.

**We thank Reviewer #3 for this important comment and agree that the terminology used to describe menstrual cycle phase should be more precise. We have revised the Methods section to state that testing occurred between days 10–15 following the onset of menstruation, based on participant self-report, rather than referring to this period as the ‘mid-follicular phase.’**

**We also acknowledge that, in the absence of objective verification e.g., hormonal assays or ovulation testing, it is not possible to confirm cycle phase, and this has now been explicitly noted as a limitation in the Discussion. We have incorporated the reviewer’s suggested reference to support this clarification.**

5. L112 : Could participants with amenorrhea be included in the study?

**We thank Reviewer #3 for this important point. Participants with amenorrhea were not included in the study. Regular menstrual cycles were required as part of the inclusion criteria, and menstrual cycle timing was determined based on self-reported cycle length and day. This has now been clarified in the Subjects section of the Methods to improve transparency.**

6. L.114 : The term “self-reported mid-follicular phase” requires clarification. It is unclear what participants were specifically asked to report and how this information was used to determine cycle phase. Did participants report the first day of their last menstruation, typical cycle length, or use any tracking method (e.g., calendar, app)?

**We thank Reviewer #3 for this helpful clarification. Menstrual cycle timing was determined based on participant self-report of (i) the first day of their most recent menstruation and (ii) their typical cycle length. This information was used to estimate current cycle day and schedule testing between days 10–15 following menstruation. This has now been clarified in the Subjects section of the Methods to improve transparency.**

7. Were participants fully informed about the protocol and the presence of caffeine and placebo?

**We thank Reviewer #3 for this important point. All participants provided written informed consent prior to enrolment and were fully informed about the general study procedures, including that the study involved caffeine supplementation and placebo conditions. However, participants were not informed of the specific condition being administered at each session, consistent with the single-blind design. This has now been clarified in the Methods section.**

8. L209 : There is no information regarding the rate of perceived exertion scale used. I assume a CR10 scale was employed, but this should be explicitly stated and referenced.

**We thank Reviewer #3 for this observation. The rate of perceived exertion (RPE) scale used in the study was the Borg CR10 scale. This has now been explicitly stated in the Methods section, and an appropriate reference has been added.**

RESULTS
9. Table 1 : Please can you include absolute torque values in addition to normalized data.

**We thank Reviewer #3 for this helpful suggestion. Absolute torque values have now been included alongside body mass-normalised values in Table 1 to improve transparency and allow for comparison with studies reporting absolute strength measures. Normalised values remain the primary outcome to account for differences in body mass.**

DISCUSSION
10. L391-400 : While the focus on female participants represents an important aspect of the study, this section currently lacks a clear connection with the results actually presented. The discussion highlights several relevant physiological considerations (e.g., hormonal modulation of CYP1A2 activity or the effects of menstrual cycle phase), but these are not discussed in relation to the data collected in the present study. The discussion would benefit from more connections, or alternatively, presenting them as avenues for future research. Furthermore, the claim that the menstrual cycle was controlled appears an overstatement. In the absence of objective verification (e.g. hormonal assays or ovulation detection), it is not possible to confirm that all participants were in the same phase of the cycle, nor that this phase was consistent across testing sessions.

**We thank Reviewer #3 for this insightful comment. We agree that the discussion of female-specific physiology should be more clearly aligned with the data presented. Accordingly, we have revised this section to better distinguish between interpretations supported by the current findings and broader physiological considerations. Specifically, we have linked the discussion of caffeine’s effects to the observed improvements in torque, time to exhaustion, and EMG amplitude, while framing hormonal influences (e.g., CYP1A2 modulation and menstrual cycle effects) more explicitly as potential explanatory mechanisms and directions for future research.**

**We have also revised the wording to avoid overstatement regarding menstrual cycle control. The manuscript now clarifies that cycle timing was standardised based on self-report rather than objectively verified, and this has been consistently acknowledged as a limitation.**

MINOR COMMENTS
INTRODUCTION
11. L31: “Athletic and neuromuscular performance follows a clear diurnal rhythm, with outputs typically lowest in the early morning and peaking in the late afternoon or evening.”
I suggest to tone down this sentence as research on circadian preferences (chronotyping), circadian variations and neuromuscular performance agrees on the influence of circadian rhythms and evening “boost” but seems also to suggest high intraindividual variability (e.g. doi: 10.3389/fspor.2024.1466050; or doi: 10.1016/j.cub.2014.12.036).

**We thank Reviewer #3 for this helpful suggestion. We agree that the original wording was overly definitive. The sentence has now been revised to better reflect inter-individual variability in circadian responses, and appropriate references have been incorporated.**

12. L59: “it a promising strategy to restore morning neuromuscular function to that of evening levels” Not sure restoring is the correct word here. Later on you used “enhanced”. It is clearer.

**We thank Reviewer #3 for this helpful suggestion. We agree that the term ‘restore’ may overstate the findings. The wording has been revised to ‘enhance’ for clarity and consistency throughout the manuscript.**

RESULTS
13. All figure : Please include detailed figure legends to facilitate the reading of the results.

**We thank Reviewer #3 for this comment. While the figure legends already contained detailed methodological information, we have revised all figure legends to further improve clarity and ensure that each figure is fully self-contained. Specifically, we have streamlined the presentation, clarified abbreviations and statistical annotations, and added brief summary statements to aid interpretation without requiring reference to the main text.**

14. I suggest to run exploratory correlation analyses to investigate whether within your sample more AM-PM temperature was associated with higher force differences between AM and PM, and if it is the case, if the same could be observed between AM placebo and AM caffeine, (similar analysis could be performed for EMG-RMS. that could strengthen the discussion on which mechanisms are most plausible to rise neuromuscular performance following caffeine ingestion in your sample vs the literature.

**We thank Reviewer #3 for this thoughtful suggestion. We agree that exploratory correlation analyses examining associations between diurnal changes in temperature, neuromuscular function, and EMG parameters could provide additional mechanistic insight. However, given the relatively small sample size and the exploratory nature of such analyses, we believe that these would be underpowered and may increase the risk of spurious associations or overinterpretation. As such, we have not included these analyses in the present manuscript. We have, however, incorporated this suggestion into the Discussion as a potential avenue for future research to further elucidate the mechanisms underpinning the observed effects.**

15. For the perspective it could be argued that it is time to model the kinetics of neuromuscular evolution in function of circadian rhythm and caffeine ingestion, similarly to what it has been done with vigilance (<https://pmc.ncbi.nlm.nih.gov/articles/PMC5103804>).

**We thank Reviewer #3 for this insightful suggestion. We agree that modelling the temporal dynamics of neuromuscular performance in relation to circadian rhythms and caffeine ingestion represents an important future direction for the field. While such approaches were beyond the scope of the present study, we have now incorporated this perspective into the Discussion, highlighting the potential for future work to adopt modelling frameworks similar to those applied in related domains such as vigilance.**
