# Supplementary material for: Caffeine ingestion improves morning neuromuscular performance to evening levels in healthy females
Source: PLoS One. 2026 May 7;21(5):e0336199. doi: 10.1371/journal.pone.0336199 (PMC13152181; doi:10.1371/journal.pone.0336199)
Supplement: Attachment — Submitted filename: Response_to_Reviewers_auresp_3.docx [file pone.0336199.s003.docx]

Thank you for the careful consideration of our manuscript and for the constructive and insightful comments provided by both the Academic Editor and Reviewer #3. We are pleased that the revisions have improved the clarity and quality of the manuscript. We have addressed all comments in detail below point by point and recorded our responses in **bold red text**. The manuscript has also been revised accordingly.

Editor Comments:

Thank you for addressing the comments of the reviewers and mine. Please:

Make data openly available. Please see journal policies here: <https://journals.plos.org/plosone/s/data-availability#loc-acceptable-data-access-restrictions>

**We thank the Editor for highlighting this point. All data underlying the findings of this study are now available in a public repository (Figshare) and are openly available at: https://doi.org/10.6084/m9.figshare.30382900**

**The Data Availability Statement in the manuscript has been updated accordingly to reflect this.**

Please add hours of recovery that separated sessions in Figure 1, Please place sessions in chronological order in table 1. Consider to reduce the opacity in the other figures to facilitate the reading of individual trajectories.

**We thank the editor for the suggestions. Figure 1 has been revised to explicitly include the recovery period before sessions. The schematic now clearly indicates the timing between trials.**

**Table 1 has been revised so that conditions are presented in chronological order (PM_PLAC_ → AM_PLAC_ → AM_CAFF_), consistent with the study design.**

**We have reduced the opacity of the bar plots across figures to improve the visibility of individual data points and trajectories. Individual observations and paired changes are now more clearly discernible.**

The reviewer raises an additional valid point: please address the reviewer last comment. In that direction please also 1) clearly state main and secondary outcomes in the manuscript methods section.

**We have clarified the primary and secondary outcomes in the Methods section. Adding the text: “The primary outcome was maximal voluntary isometric contraction (MVIC) torque. Secondary outcomes included time to exhaustion, EMG-derived variables (RMS and MDF), rating of perceived exertion, and tympanic temperature.”**

2) I suggest framing the manuscript a bit more cautiously, as an improvement of ~20-30% is incredibly high and some other factors might occurred. Please when comparing results with the current literature, compare magnitude (e.g. your ~30% improvement vs the X% improvement of other studies).

**We thank the Editor for this important point. The manuscript has been revised to adopt a more cautious interpretation of the findings. Specifically, we have avoided causal or overstated language throughout the manuscript and now frame the results in terms of between-condition differences. In addition, we have included a statement in the Discussion acknowledging that the magnitude of the observed effects (~20–30%) is relatively large and may reflect additional contributing factors beyond caffeine ingestion alone.**

One last comment, as I occurred to notice: L209 mention you performed that you measured MVIC after 2 min "to avoid fatigue". However this makes little sense since it is the goal was to test for reduced fatigability after caffeine ingestion. Please consider reformulating in something like "to assess longer-term force suppression" or something similar.

**We thank the Editor for this important clarification. The wording has been revised to remove the implication that the rest interval was used ‘to avoid fatigue,’ ensuring a more accurate and neutral description of the protocol.**


Reviewers' comments:

Reviewer #3: The revised manuscript reflects a thorough and thoughtful response to the reviewers’ feedback. The revisions have meaningfully enhanced the paper’s quality, especially in terms of clearer methodology, improved transparency in the description of procedures, and a more explicit discussion of the study’s limitations.

**We thank the reviewer for their positive evaluation and are pleased that the revisions have improved the clarity and overall quality of the manuscript.**

However, since MVIC was the primary outcome and was assessed only after caffeine ingestion, the manuscript should frame interpretations in terms of condition effects rather than implying a direct pre–post effect of caffeine within the same session. This distinction should be stated more clearly in the main text, not only in the limitations section.

**We thank the reviewer for this important point. We agree that, given the study design, MVIC outcomes should be interpreted as between-condition effects rather than within-session pre–post caffeine effects. The manuscript has been revised accordingly, with language clarified in both the Results and Discussion sections to consistently reflect this distinction.**
